# Supplementary material for: Emergence of Cfr-Mediated Linezolid Resistance among Livestock-Associated Methicillin-Resistant Staphylococcus aureus (LA-MRSA) from Healthy Pigs in Portugal
Source: Antibiotics (Basel). 2022 Oct 19;11(10):1439. doi: 10.3390/antibiotics11101439 (PMC9598761; doi:10.3390/antibiotics11101439)
Supplement: Supplementary file 1 [file antibiotics-11-01439-s001.zip › antibiotics-1943577-supplementary.pdf]

Table S1 - Antibiotic resistance patterns of MRSA isolates.

Antibiotic Resistance Patterns	N. of isolates	% MDR isolates
FOX, CHL, CIP, CLI, ERY, KAN, PEN, SYN, STR, TET, TMP	1	0.6
FOX, CHL, CIP, CLI, ERY, PEN, SYN, TET, TIA	7	4.2
FOX, CHL, CIP, CLI, ERY, PEN, SYN, TET, TIA, TMP	1	0.6
FOX, CHL, CIP, CLI, ERY, PEN, TET	1	0.6
FOX, CHL, CIP, CLI, PEN, TET, TIA	7	4.2
FOX, CHL, CIP, CLI, PEN, TET, TIA, TMP	1	0.6
FOX, CHL, CIP, PEN, STR, TET	1	0.6
FOX, CHL, CIP, PEN, TET	2	1.2
FOX, CHL, CLI, ERY, GEN, KAN, PEN, SYN, STR, TET, TIA	1	0.6
FOX, CHL, CLI, ERY, GEN, KAN, PEN, SYN, TET, TIA	1	0.6
FOX, CHL, CLI, ERY, GEN, KAN, PEN, SYN, TET, TIA, TMP	2	1.2
FOX, CHL, CLI, ERY, KAN, PEN, SYN, TET, TIA	1	0.6
FOX, CHL, CLI, ERY, LZD, PEN, SYN, TET, TIA, TMP	2	1.2
FOX, CHL, CLI, ERY, PEN, SYN, TET, TIA	4	2.4
FOX, CHL, CLI, GEN, KAN, PEN, STR, TET, TIA, TMP	1	0.6
FOX, CHL, CLI, LZD, PEN, SYN, TET, TIA	1	0.6
FOX, CHL, CLI, PEN, TET, TIA	6	3.6
FOX, CHL, CLI, PEN, TET, TIA, TMP	1	0.6
FOX, CHL, KAN, PEN, TET, TMP	2	1.2
FOX, CHL, PEN, TET	3	1.8
FOX, CIP, CLI, ERY, FUS, PEN, TET, TIA, TMP	1	0.6
FOX, CIP, CLI, ERY, GEN, KAN, PEN, SYN, TET, TIA, TMP	2	1.2
FOX, CIP, CLI, ERY, GEN, KAN, PEN, SYN, TET, TMP	1	0.6
FOX, CIP, CLI, ERY, GEN, KAN, PEN, TET, TMP	2	1.2
FOX, CIP, CLI, ERY, KAN, PEN, SYN, TET, TIA, TMP	1	0.6
FOX, CIP, CLI, ERY, PEN, SYN, STR, TET, TIA	3	1.8
FOX, CIP, CLI, ERY, PEN, SYN, TET, TIA	2	1.2
FOX, CIP, CLI, ERY, PEN, SYN, TET, TIA, TMP	7	4.2
FOX, CIP, CLI, ERY, PEN, SYN, TET, TMP	1	0.6
FOX, CIP, CLI, KAN, PEN, TET, TIA, TMP	1	0.6
FOX, CIP, CLI, PEN, STR, TET, TIA	2	1.2
FOX, CIP, CLI, PEN, TET, TIA, TMP	6	3.6
FOX, CIP, PEN, STR, TET	2	1.3
FOX, CIP, PEN, TET	1	0.6
FOX, CIP, PEN, TET, TMP	3	1.8
FOX, CLI, ERY, FUS, GEN, KAN, MUP, PEN, SYN, RIF, STR, SMX, TET, TIA, TMP	1	0.6
FOX, CLI, ERY, FUS, GEN, KAN, MUP, PEN, SYN, STR, SMX, TET, TIA	1	0.6
FOX, CLI, ERY, GEN, KAN, PEN, SYN, TET, TIA, TMP	1	0.6

FOX, CLI, ERY, KAN, PEN, SYN, TET, TIA	2	1.2
FOX, CLI, ERY, KAN, PEN, SYN, TET, TIA, TMP	5	3.0
FOX, CLI, ERY, PEN, STR, TET	2	1.2
FOX, CLI, ERY, PEN, SYN, STR, TET, TIA	1	0.6
FOX, CLI, ERY, PEN, SYN, TET	1	0.6
FOX, CLI, ERY, PEN, SYN, TET, TIA	17	10.3
FOX, CLI, ERY, PEN, SYN, TET, TIA, TMP	27	16.4
FOX, CLI, ERY, PEN, SYN, TET, TMP	1	0.6
FOX, CLI, ERY, PEN, TET	2	1.2
FOX, CLI, ERY, PEN, TET, TMP	2	1.2
FOX, CLI, GEN, KAN, PEN, TET, TIA, TMP	1	0.6
FOX, CLI, KAN, PEN, TET, TIA	1	0.6
FOX, CLI, PEN, SYN, TET, TIA	5	3.0
FOX, CLI, PEN, SYN, TET, TIA, TMP	1	0.6
FOX, CLI, PEN, TET, TIA	4	2.4
FOX, CLI, PEN, TET, TIA, TMP	7	4.2
FOX, PEN, TET	4	*
FOX, PEN, TET, TMP	2	1.2

*Not multidrug

FOX, cefoxitin; CLI, clindamycin; PEN, penicillin; KAN, kanamycin; SYN, quinupristin-dalfopristin; TET, tetracycline; TIA, tiamulin; TRI, trimethoprim; STR, streptomycin; ERI, erythromycin; MUP, mupirocin; SMX, sulphamethoxazole; FUS, fusidic acid; GEN, gentamicin; RIF, rifampicin.
